# Post-training Inactivation of the Anterior Thalamic Nuclei Impairs Spatial Performance on the Radial Arm Maze

**DOI:** 10.3389/fnins.2017.00094

**Published:** 2017-03-06

**Authors:** Ryan E. Harvey, Shannon M. Thompson, Lilliana M. Sanchez, Ryan M. Yoder, Benjamin J. Clark

**Affiliations:** ^1^Department of Psychology, University of New MexicoAlbuquerque, NM, USA; ^2^Department of Psychology, Indiana-Purdue UniversityFort Wayne, IN, USA

**Keywords:** navigation, head direction, spatial, limbic, hippocampus

## Abstract

The limbic thalamus, specifically the anterior thalamic nuclei (ATN), contains brain signals including that of head direction cells, which fire as a function of an animal's directional orientation in an environment. Recent work has suggested that this directional orientation information stemming from the ATN contributes to the generation of hippocampal and parahippocampal spatial representations, and may contribute to the establishment of unique spatial representations in radially oriented tasks such as the radial arm maze. While previous studies have shown that ATN lesions can impair spatial working memory performance in the radial maze, little work has been done to investigate spatial reference memory in a discrimination task variant. Further, while previous studies have shown that ATN lesions can impair performance in the radial maze, these studies produced the ATN lesions prior to training. It is therefore unclear whether the ATN lesions disrupted acquisition or retention of radial maze performance. Here, we tested the role of ATN signaling in a previously learned spatial discrimination task on a radial arm maze. Rats were first trained to asymptotic levels in a task in which two maze arms were consistently baited across training. After 24 h, animals received muscimol inactivation of the ATN before a 4 trial probe test. We report impairments in post-inactivation trials, suggesting that signals from the ATN modulate the use of a previously acquired spatial discrimination in the radial-arm maze. The results are discussed in relation to the thalamo-cortical limbic circuits involved in spatial information processing, with an emphasis on the head direction signal.

## Introduction

The ability to navigate depends on tracking moment-to-moment changes in directional orientation and spatial location when moving from one place to another. In rodents, spatial representations of an environment appear to be provided by place cells, head direction cells, and grid cells located in the hippocampus and limbic brain regions (McNaughton et al., 2006; Taube, 2007; Moser et al., 2008). Place cells are hippocampal neurons that fire as a function of an animal's location in an environment, and parahippocampal grid cells fire in multiple spatial locations forming a hexagonal grid-like pattern. Head direction cells are modulated by an animal's directional orientation and are found throughout the classic Papez circuit, including the anterior thalamic nuclei (ATN; anterodorsal, anteroventral, and anteromedial thalamus), postsubiculum, and other limbic cortical regions (Taube, 1995; Aggleton et al., 2010; Tsanov et al., 2011; Jankowski et al., 2015; Wilber et al., 2015; Clark and Harvey, 2016). The directional orientation of head direction cells are strongly influenced by allothetic spatial stimuli such as environmental landmarks, but in the absence of stable environmental features, the orientation of head direction cells can be maintained by self-movement cues such as vestibular, proprioceptive, and motor stimuli (Clark and Taube, 2009; Shinder and Taube, 2011; Yoder et al., 2011a, 2015; Clark et al., 2012; Clark and Taube, 2012).

The role of head direction cell activity in spatial navigation is poorly understood, but one hypothesis states that directional signals conveyed by the limbic thalamus may influence the orientation and stability of hippocampal place cell signals in relation to environmental landmarks (Yoganarasimha et al., 2006; Yoder et al., 2011b). Supporting this view, lesions of the postsubiculum significantly impair the stability and landmark control of hippocampal place cells (Calton et al., 2003), and disrupt spatial performance on a radial arm maze (Taube et al., 1992). In contrast, however, lesions of the ATN fail to produce similar impairments in hippocampal place cell activity, suggesting a possible dissociation in function between the ATN and other regions within head direction cell circuitry (Calton et al., 2003). An additional hypothesis regarding the relationship between head direction cells and hippocampal processing is based on recent work demonstrating a tendency for place cells to establish unique spatial firing patterns in radially oriented environments or in environments with opposed directions, but to form similar firing patterns in parallel environments with similar directional orientations (Fuhs et al., 2005; Derdikman et al., 2009; Spiers et al., 2015; Grieves et al., 2016). This observation points to the possibility that representations of directional orientation might facilitate the generation of distinct spatial representations in radial environments (Wood et al., 2016). Accordingly, lesioning or inactivating some or all ATN before training impairs performance in radially oriented environments (Aggleton et al., 1996; Alexinsky, 2001; Mair et al., 2003; Mitchell and Dalrymple-Alford, 2005, 2006). However, little is known regarding the influence of ATN damage on post-acquisition performance in the radial maze. Further, previous studies have utilized procedures that largely assessed spatial working memory in which animals were typically exposed to a radial maze in which all of the arms were baited, and spatial errors were scored as returns to previously visited arms within a given session (e.g., Beracochea et al., 1989; Aggleton et al., 1996; Mair et al., 2003; Mitchell and Dalrymple-Alford, 2006). This type of assessment tests whether animals can retain information regarding their previous arm selections during a session (spatial working memory), but does not require the animal to retain spatial information across daily test sessions (spatial reference memory). It therefore remains unclear whether the information conveyed by the ATN is necessary for the pre-training or post-training performance, or both, on radial tasks that require spatial discrimination within and between daily test sessions.

Some recent evidence points to the possibility that the head direction cell signal may contribute to navigational performance in radial tasks. Using a radial arm maze discrimination task, Yoder and Kirby (2014) reported spatial deficits in genetically modified mice lacking functional otolith organs, an animal model previously shown to have impaired ATN head direction cell signals (Yoder and Taube, 2009). A caveat to this interpretation is that vestibular dysfunction can affect brain processes other than the head direction signal (Russell et al., 2003a,b, 2006; Horii et al., 2004; for reviews, see Smith et al., 2005; Hitier et al., 2014; Yoder and Taube, 2014), and whether otoconia-deficient mice's radial maze deficits resulted from disrupted head direction signal processing remains to be tested. Given the ATN's crucial role in conveying the head direction signal to limbic and hippocampal regions, we assessed the role of the ATN in performance in a similar radial arm maze task in which two maze arms were consistently baited across daily training sessions; thus, animals were required to distinguish baited from unbaited arms by using the relationship between distal spatial cues and reward locations.

## Methods

### Subjects

Subjects were 12 male hooded Long-Evans rats (Harlan, Indianapolis, IN) that were ~160 days of age at the beginning of the experiments. All animals were pair-housed in plastic cages on a reverse 12 h light:dark cycle with food and water available *ad libitum*. During habituation training and experiments, rats were placed on a restricted food diet to maintain 90% of their *ad libitum* weight. Rats were given access to water *ad libitum*. The Institutional Animal Care and Use Committee at the University of New Mexico approved all procedures for the studies reported here.

### Surgery

Twelve rats were surgically implanted with custom fabricated bilateral cannula that targeted the anterior thalamus. The custom cannulas were made of two 26-gauge stainless steel outer cannula and 33-gauge inner dummy cannula. Animals were anesthetized with isoflurane and placed in a stereotaxic frame with atraumatic ear bars. The head was adjusted in the frame to achieve flat skull coordinates. Anesthesia was maintained via an inhalation nose cone affixed to the mouth bar on the frame. Lidocaine (2%) was used as a local anesthetic underneath the skin above the skull. Under sterile conditions, a midline incision was made, and the skull exposed. The outer cannula were targeted just above the ATN such that the inner infusion cannula, which protruded ~1 mm below the outer cannula, would be centrally placed within the ATN at the following coordinates relative to bregma: anterior-posterior −1.74 mm, medial-lateral 1.25 mm (2.48 mm between two cannula), dorsal-ventral (DV) −5.23 mm (DV coordinate measured from skull surface) and were held in place using dental acrylic. Coordinates were based on plates from Paxinos and Watson (1998) and previous histological assessment. After completion of the implantations, the skin was cleaned and sutured. The rats were given subcutaneous injections of buprenex (0.03 mg/ml concentration and a 0.1 mg/kg dosage) right after surgery and once a day for 2 days. Following surgery, rats were single-housed to prevent damage to the implant. All rats were given 7 days to recover with unlimited access to food and water, followed by 7 days of food restriction prior to returning to the experiment.

### Radial arm maze

The radial arm maze consisted of eight black Plexiglas arms (each 40.1 × 9.30 cm, separated by 45° from each other) that radiated out from a center platform (25 cm in diameter). One recessed reward cup was located on a platform (20 × 30 cm) at the distal end of each arm. The maze was located near a corner of a testing room with many extra-maze cues, including a sink, filing cabinet, chair, and wall posters. A transparent plastic cylinder (25 cm in diameter) located in the center of the maze was used to restrict the rats to that region of the maze before the initiation of a training trial. A camera was positioned above the maze and digital videos were obtained for each testing session for off-line analysis.

### Habituation trials

Rats first underwent 10 min habituation training trials on the radial arm maze over 3 consecutive days. During habituation, all of the recessed cups were baited with a food reward (quarter piece of dry cereal). In a trial, rats were first placed in the transparent cylinder located at the center of the maze for 15–30 s, followed by the removal of the cylinder, thereby allowing the rat to explore the maze and consume food from the food cups for the remaining time.

### Acquisition trials

Acquisition training occurred over 11 days in blocks of four trials per day. In this phase of training, only two of the eight arms (separated by 135°) were baited with a food reward. The spatial relationship between the baited maze arms and the room cues was maintained throughout training and the baited/un-baited arm configuration was counterbalanced across rats. At the beginning of each trial, the rat was placed in the cylinder in the center of the maze, where it remained for 15–30 s before starting the trial. To discourage the use of the experimenter as a cue, the direction in which the rat was placed in the cylinder was counterbalanced over trials. After the 15–30 s had elapsed, the rat was released and allowed to freely investigate the maze and search for the baited arms. Trials were terminated when the animal either located the baited arms and consumed the food reward, or after 5 min had elapsed. Once rats located and consumed the reward, they were returned to a transport cage for ~1 min while the entire maze was cleaned with a non-toxic cleaning solution. The maze was cleaned and rotated 180° at the end of each day to discourage the use of intra-maze cues (e.g., local features and/or odors) between days.

### Inactivation probe

After acquisition, rats completed two probe trial blocks on the RAM using a within-subjects cross design (Law and Smith, 2012; Stackman et al., 2012). On day 12, half of the rats received bilateral intracranial infusion of muscimol into the ATN (0.25 ul at a concentration of 0.25 ug/ul; Tocaris Bioscience), while the remaining rats received infusions of saline (0.25 ul at a concentration of 0.9%). This volume of muscimol and saline was selected to maintain consistency with previous studies. Specifically, Stackman et al. (2012) estimated that 0.25 ul of muscimol infused within the ATN was confined within 0.25–0.30 mm from the injection site, and Allen et al. (2008) estimated that 0.5 ul of muscimol infused within the dorsomedial prefrontal cortex had a spread that was confined within 0.5–1.0 mm from the injection site. In consideration of previous studies, we estimated that the volume of 0.25 ul of muscimol would likely be confined to the ATN (i.e., mostly the anterior dorsal thalamus and partially within the anterior ventral thalamus). Muscimol was used because it avoids the concerns of lidocaine, which also affect fibers of passage. Infusions were administered by first gently restraining the rats, removing the dummy cannula, and then inserting the bilateral 33-gauge infusion cannula. Infusions were performed through two 10 uL Hamilton syringes held in a Harvard Apparatus “22” syringe pump (Harvard Apparatus, MA). The infusions were delivered at a rate of 0.167/min for 1.5 min, infusion cannula remained in place for 30 s after the infusions, and dummy cannula were then re-inserted. Each rat was returned to a transport cage for 30 min before being transported to the radial arm maze for the probe test. On day 13, all rats completed another trial block of radial arm maze testing, and on day 14, rats received a second intracranial infusion of muscimol or saline followed by a trial block in the radial arm maze using the same methods described above. On day 14, treatment conditions were reversed such that all rats received both muscimol and saline infusions.

### Scoring and data analysis

To provide a comparison to previous studies, we utilized measures traditionally used to assess spatial memory on a radial maze, i.e., spatial reference memory and spatial working memory (Olton and Papas, 1979; Yoder and Kirby, 2014). It is important to note, however, that our use of the term “memory” in these measures does not imply that the observed deficits are a consequence of memory dysfunction *per-se*, but could simply reflect general impairments in navigation.

Performance measures included the percentage of correct trials, the number of errors, and search latency calculated for each animal during acquisition and probe testing, as previously described by Yoder and Kirby (2014). Briefly, an arm choice was counted when all four of the rat's paws crossed the threshold of an arm. A correct choice was counted only if the rat approached and ate from a baited food cup. Percentage of correct arm choices was calculated as the number of correct choices divided by the total number of arm entries within a trial. Error trials were categorized into three subtypes: spatial reference memory (RM) errors, spatial working memory-correct (WM-C) errors, and spatial working memory-incorrect (WM-I) errors. RM errors occurred when a rat first entered an unbaited arm, or if they entered a baited arm without approaching and eating from the food cup. This partial entry was classified as an RM error because the rat had made a choice (arm entry) that did not meet the criteria to be classified as a correct choice. WM-C errors occurred when a rat re-entered an arm that had been previously baited. WM-I errors occurred when a rat re-entered an arm that never contained a reward. Latency was measured as the time elapsed from the beginning to the end of each trial. All measures were averaged across each trial block.

Video records were evaluated for the search strategy used by rats during acquisition and probe testing. Two strategies were identified based on previous descriptions: spatial and serial subtypes (Hodges, 1996). A search path was spatial if an animal's last two arm choices were baited. A serial strategy occurred when an animal first visited a baited arm and then subsequently visited arms in a clockwise or counter clockwise fashion until they reached the second baited arm. However, we categorized a trial that included adjacent and non-adjacent arm visits as a “mixed” strategy because a true serial strategy was not observed throughout the experiment. Finally, video records from probe tests were scored for behaviors reflecting door exploration. These movements are similar to the horizontal head scanning movements previously described as “vicarious trial-and-error” (Tolman, 1939) or “microchoices” (Brown and Cook, 1986), and are characterized as side-to-side head movements directed toward the entry point of maze arms, but occur without an explicit arm choice (Brown and Cook, 1986; Bimonte and Denenberg, 2000; Bett et al., 2015; Redish, 2016). Door exploration was scored when animals paused near the entry of a maze arm and appeared to investigate with at least their nose passing within ~2.5 cm of the threshold of the arm. If an animal crossed the threshold with all four paws, a door exploration movement was not counted.

Acquisition measures were subjected to repeated measures analysis of variance (ANOVA) with trial block as within subject factors. A multivariate repeated measures ANOVA was used to test search strategy performance with strategy as between subject factors and trial block as within subject factors. For the probe test, behavioral measures were subjected to paired *t*-tests (two-tailed). ANOVAs and *t*-tests were conducted using SPSS (23.0, SPSS Inc., Armonk, NY). Effect sizes for ANOVAs and *t*-tests were calculated using Cohen's d (d) and partial eta squared (η^2^), respectively.

### Histology

At the completion of testing, rats were deeply anesthetized with sodium pentobarbital and were then transcardially perfused with saline, followed by a 4% formalin solution. The brains were removed from the skull and were post-fixed in 4% formalin for 24 h. The brains were then cryoprotected in a 30% sucrose solution for at least 24 h. A cryostat was used to cut 40 um coronal sections through the ATN. Each section was mounted on glass microscope slides, dried, and stained with crystal violet before being cover-slipped. Bilateral placement of infusion cannula was examined under light microscopy.

## Results

### Histology

**Figure 1:**
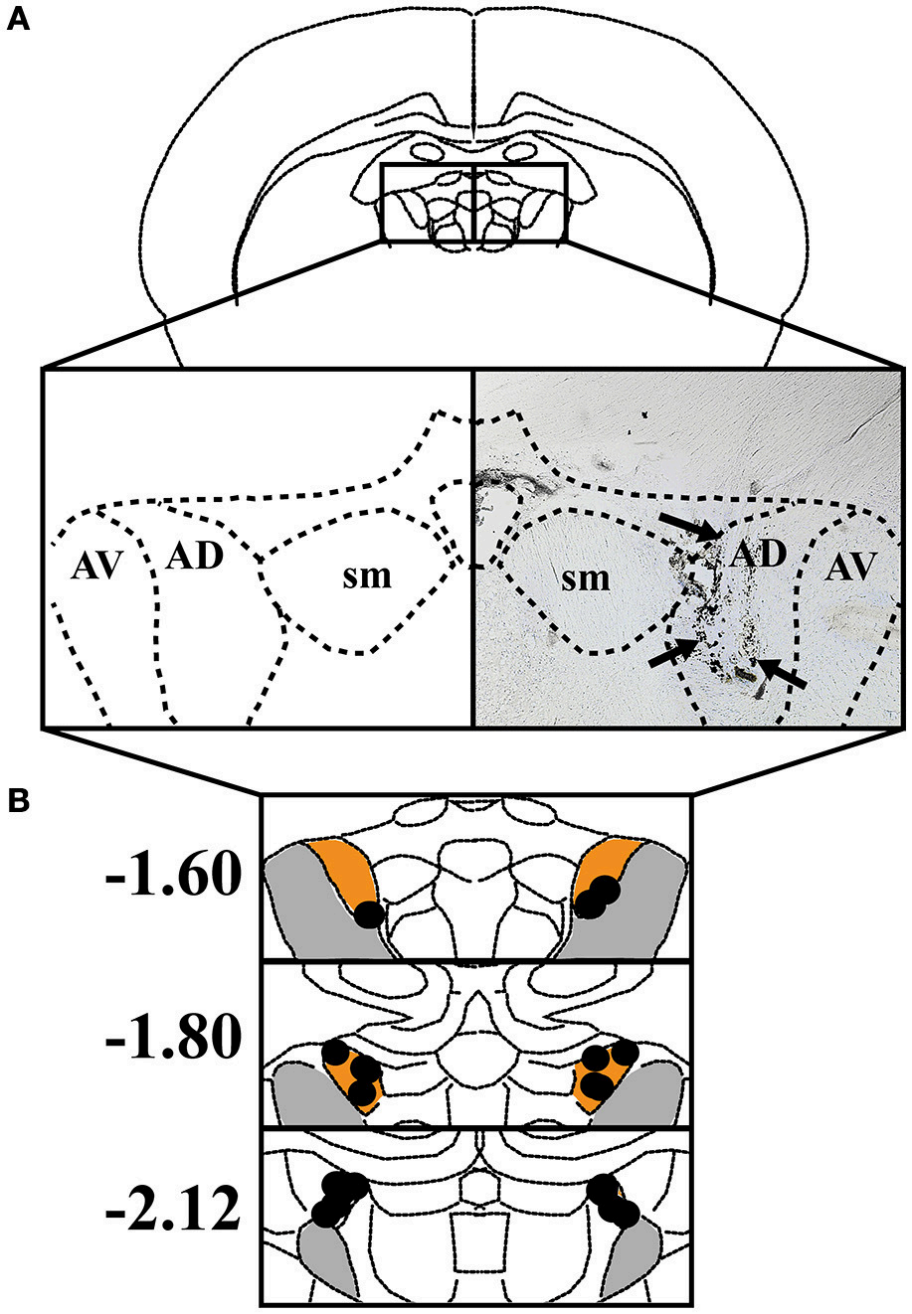
**(A)** Left: The anterodorsal thalamic nuclei (AD), anteroventral thalamic nuclei (AV), and stria medullaris (SM) are shown in a coronal view. Right: Representative coronal section depicting an infusion track through the ATN. Black arrowheads indicate track of infusion cannula. **(B)** The individual placements of infusion sites are indicated with black circles and presented against coronal views of the ATN at three rostral-caudal levels (in mm relative to bregma). Orange represents AD and gray represents AV.

Histological analysis confirmed that the majority of cannula were placed within the ATN, particularly the anterodorsal and anteroventral subnuclei (*n* = 10). In one case, however, cannula placement was observed in the habenula, and in a second case, bilateral cannula were located within the boundaries of the mediodorsal thalamus. Because infusions in the latter two rats included adjacent subcortical regions, the data from these animals were excluded from further analysis. Figure 1 shows the results of histological analysis from the remaining 10 rats included in the behavioral analyses below. In comparison with our cannula placements and the previous literature on muscimol spread, we estimate that muscimol infusions were limited to the anterodorsal and anteroventral thalamic nuclei.

### Acquisition

**Figure 2:**
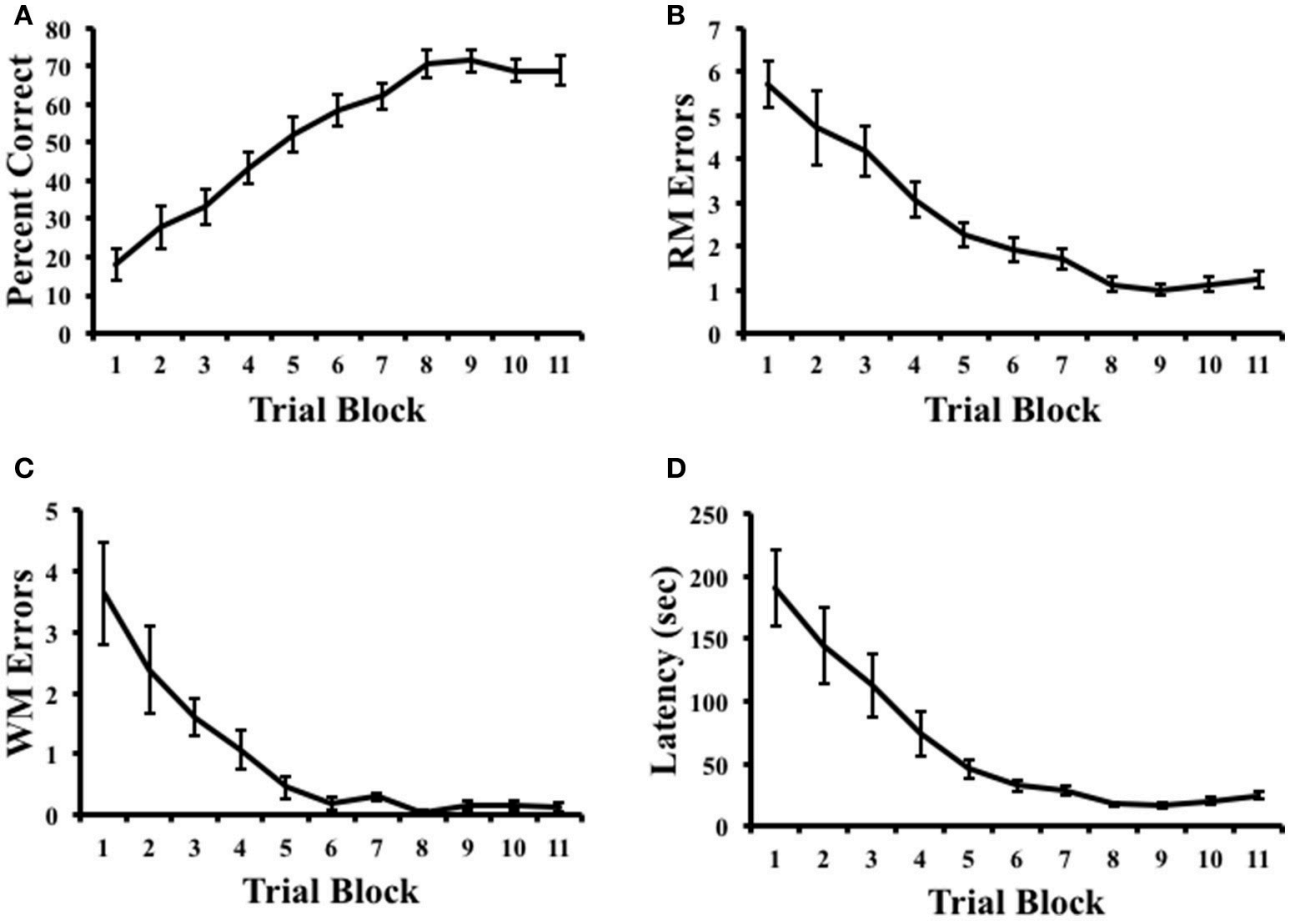
**Results of radial arm maze task acquisition. (A)** Percentage of correct arm choices increased over trial blocks. **(B,C)** Spatial reference memory (RM) and spatial working memory (WM) errors decreased across trial blocks. **(D)** Latency to complete the task decreased across trial blocks. Mean ± SEM.

Figure 2 plots the percentage of correct trials, RM errors, WM errors, and latency across radial arm maze training. A repeated measures ANOVA on pre-inactivation performance indicated that rats showed increasing measures of percent correct, [*F*_(10, 90)_ = 27.65, *p* < 0.001, η^2^ = 0.75], reduced RM errors, [*F*_(10, 90)_ = 27.08, *p* < 0.001, η^2^ = 0.71], reduced WM errors, [*F*_(10, 90)_ = 22.23, *p* < 0.001, η^2^ = 0.54], and decreasing measures of latency, [*F*_(10, 90)_ = 17.158, *p* < 0.001, η^2^ = 0.66], suggesting that animals had learned the task by the end of training. Indeed, measures of the percentage of correct trials were significantly above chance performance (25%) on the final day of training [Mean ± SEM: 68.92 ± 3.77%; *t*_(9)_ = 11.67, *p* < 0.001].

### Inactivation probe trials

**Figure 3:**
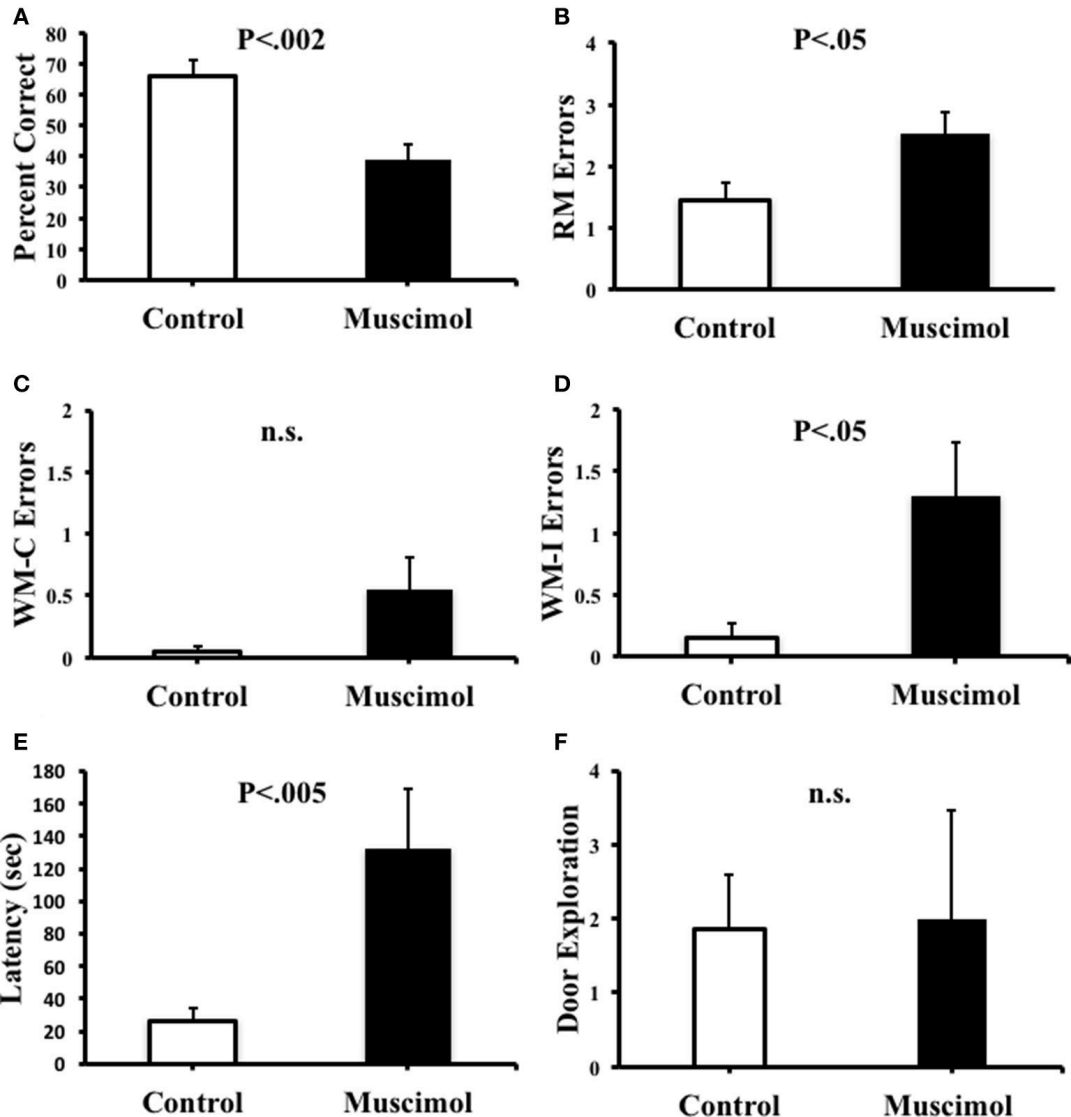
**(A)** Percentage of correct arm choices was higher in control animals than in muscimol animals. **(B)** Spatial reference memory (RM) error were significantly greater in muscimol animals than in control animals. **(C)** Spatial working memory-correct (WM-C) errors did not significantly differ between groups, but note that muscimol animals trended toward more WM-C errors compared to controls. **(D)** Spatial working memory-incorrect (WM-I) errors were significantly greater in muscimol animals than in control animals. **(E)** Latency to complete the task was significantly greater in muscimol animals than in control animals. **(F)** Muscimol and control animals made a similar total number of door exploration movements per trial. Mean + SEM.

Figure 3 plots the percentage of correct trials, RM errors, WM-C errors, WM-I errors, latency, and door explorations following the inactivation of ATN. In the probe trials following intracranial infusions, animals that received muscimol treatment demonstrated decreases in the accuracy of selecting the correct maze arms. This observation was confirmed by a significant reduction in the overall percentage of correct trials [*t*_(9)_ = 4.57, *p* = 0.001, *d* = 1.65], an increase in the number of RM errors [*t*_(9)_ = −2.31, *p* = 0.046, *d* = −1.04], and an increase in search latency [*t*_(9)_ = −4.31, *p* = 0.002, *d* = −1.26]. During the probe trials, RM errors in which animals entered correct arms but did not consume the food rewards were only noted in one muscimol infused animal during a single session. Due to this low frequency, this type of error was grouped with RM errors in which animals initially entered incorrect arms. We also observed a tendency for animals with muscimol infusions to perseverate their searches toward previously visited arms. Notably, in some cases, animals would alternate between two maze arms for up to 19 consecutive choices. The adoption of perseverative behavior by muscimol infused animals is captured by measures of the number of WM errors, which show a significant increase in the muscimol group compared to controls [WM-C: *t*_(9)_ = −2.72, *p* = 0.024, *d* = −1.15]. On average, muscimol infusions tended to increase the number of WM-C errors (0.55 ± 0.26 errors/trial) compared to saline infusions (0.05 ± 0.03 errors/trial), however, this difference failed to reach significance [*t*_(9)_ = −1.84, *p* = 0.098]. It is noteworthy that animals in the muscimol group showed a greater tendency to perseverate choices toward incorrect arms (1.30 ± 0.43 errors/trial) compared to correct arms (0.55 ± 0.26 errors/trial), further indicating that muscimol administration to the ATN resulted in a general failure in directing their movements toward the arms that were reinforced during training (i.e., spatial reference memory). Notably, the effect size of WM-C memory errors was slightly higher than the effect size of RM errors, which may be due to the restricted total amount of RM errors an animal can perform (one to eight errors), while the total amount of WM errors an animal can perform is only restricted by the duration of the session. Given this consideration, it is reasonable that a spatial deficit would result in a greater difference in WM errors than in RM errors on a radial maze. Analysis of the number of RM errors across the four post-inactivation trials also failed to indicate a reduction in the number of errors [muscimol: *F*_(3, 27)_ = 0.21, *p* = 0.89; control: *F*_(3, 27)_ = 0.16, *p* = 0.22], indicating a persistent impairment across probe testing.

We addressed the possibility that the impaired spatial performance by muscimol infused rats described above was due to an inability to execute the appropriate movements to guide behavior. We therefore quantified the number of door exploration movements made by rats after intracranial infusions. On average, we observed that control (1.85 ± 2.34 door explorations/trial) and muscimol (2.00 ± 4.65 door explorations/trial) animals performed a similar rate of door exploration per trial, [*t*_(9)_ = −0.12, *p* = 0.91]. We reasoned, however, that a general measure of door exploration by trial might be confounded by the fact that muscimol animals spent significantly more time on the maze per trial than control rats (see Figure 3E). A disproportionate amount of time on the maze would possibly allow additional time to perform door exploration. We therefore normalized the number of door explorations for each rat by the time spent in the center of the maze (the only region of the maze that door exploration can be performed). This analysis revealed that muscimol animals made a slightly greater number of door explorations/s compared to controls (muscimol: 1.17 ± 0.46; controls: 0.71 ± 0.19); however, this mean difference failed to reach statistical significance [*t*_(9)_ = −1.00, *p* = 0.34].

**Figure 4:**
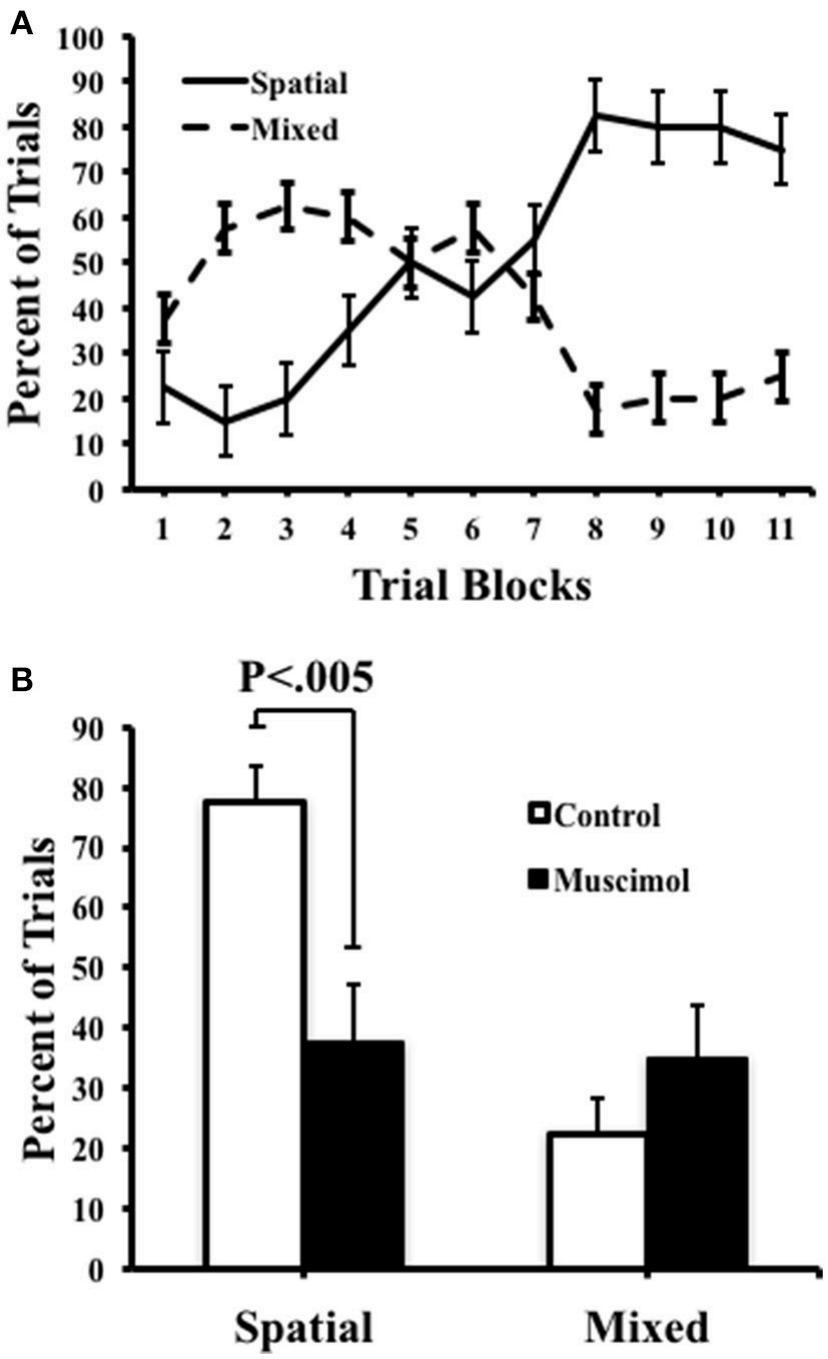
**Control and muscimol animals favored different search strategies during probe sessions. (A)** Animals favored a mixed search strategy during the early trial blocks, but ultimately favored a spatial search strategy by the end of acquisition. **(B)** During probe trials, control animals favored a spatial search strategy while muscimol animals had no preferred search strategy. Mean + SEM.

We quantified the number of spatial, serial, and mixed search strategies expressed by rats during task acquisition as well as in the probe trials (Figure 4). As expected, during training, we observed a significant interaction between strategies [*F*_(1, 9)_ = 15.00, *p* = 0.004, η^2^ = 0.63] with an increase in the number of spatial searches [*F*_(10, 90)_ = 13.27, *p* < 0.001, η^2^ = 0.60], and a corresponding decrease in mixed strategies [*F*_(10, 90)_ = 4.36, *p* ≤ 0.001, η^2^ = 0.33]. It was notable, however, that we failed to observe the use of serial strategies throughout testing, suggesting that the task demands in the present study favored spatial solutions rather than non-spatial serial behavior. Muscimol infusions resulted in a significant reduction in the percentage of spatial strategies [*F*_(1, 39)_ = 10.19, *p* = 0.003, η^2^ = 0.22], suggesting that the use of a spatial search strategy involves signals processed by ATN. A corresponding increase in the number of mixed behavioral search strategies was observed after muscimol infusions (muscimol 35.0 ± 8.97; control 22.5 + 6.03), however this mean difference failed to reach significance [*t*_(9)_ = −0.832, *p* = 4.27].

In summary, probe trials revealed that saline injection into ATN failed to disrupt navigation to the arms that were baited during acquisition, whereas muscimol injection markedly disrupted task performance. Because only two of eight arms were baited for each trial, a spatial search strategy afforded the most efficient way to navigate among the baited arms. Post-training inactivation of the ATN thus disrupted the use of a spatial search strategy on the radial arm maze discrimination task.

## Discussion

The results of the present study demonstrated clear deficits in spatial discrimination in the radial arm maze following inactivation of the ATN (see Figure 3). Specifically, animals treated with muscimol failed to accurately select the two arms of the radial maze that were consistently rewarded over 11 days of pre-training, as indicated by a significant increase in the number of RM errors and decrease in the percentage of correct trials during the probe test (see Figures 3A,B). Further, muscimol inactivation produced a greater number of WM errors which appear to be due, in part, to an increase in the number of perseverative entries into incorrect arms (see Figure 3D). A similar increase in perseverative behaviors has been observed after electrolytic lesions of the ATN (Sziklas and Petrides, 1999) and an increase in working memory errors has been observed after neurotoxic lesions of the ATN (e.g., Aggleton et al., 1996; Mair et al., 2003; Mitchell and Dalrymple-Alford, 2006). Collectively, these observations suggest that inactivated animals tended to make errors toward non-rewarded arms (i.e., RM errors), and these errors sometimes perseverated throughout probe testing (i.e., WM-I errors).

Previous studies investigating the relationship between the ATN and radial maze performance have typically used procedures in which animals are exposed to a maze with all of the arms are baited, and errors are counted when animals return to previously visited arms. Dubreuil et al. (2003) reported that rats in this task variant tend to serially sample maze arms suggesting that non-spatial procedural strategies can be favored. In the present study, we utilized a radial maze procedure in which in which two maze arms were consistently baited in each daily training session; thus, the animal was required to learn a consistent relationship among spatial cues and the reward locations. The results of the present study support the conclusion that animals in this radial maze variant learned these spatial relationships by demonstrating a significant tendency to direct movements toward the reward arms by the end of training (i.e., a spatial strategy; see Figure 4A). In contrast, the occurrence of serial search strategies was virtually non-existent throughout acquisition and probe trials further suggesting that the current radial arm maze task promotes spatial search strategies. Additionally, ATN inactivated animals used a spatial search strategy at a lower rate than control animals, supporting the conclusion that the ATN contributes to spatial discrimination in the radial arm maze.

Alexinsky (2001) utilized a similar procedure to investigate the relationship between the ATN and spatial behavior and radial arm maze performance. In this study, the author reported that animals with neurotoxic lesions of the ATN were impaired in radial arm maze performance after extensive pre-lesion training on the task. It is notable, however, that the post-surgical performance of control animals was also relatively poor compared to pre-surgical performance. A possible explanation for this finding is that the 2-week delay between pre and post-surgery trials may have been sufficient to induce spatial reference memory impairments. Thus, in the present study, we avoided this issue by surgically cannulating animals before training in the radial maze and using muscimol infusion procedures which allowed rapid inactivation of the ATN shortly (24 h) after pre-training. Further, given that there was no surgical recovery interval between acquisition and retention testing, it is unlikely that neural compensation and time-dependent pathological changes in other regions influenced performance (Jenkins et al., 2004; Dumont et al., 2012). However, we cannot exclude the possibility that changes in other neural signals that involve the ATN, such as those involved in contextual fear conditioning (de Lima et al., 2017), contributed to the observed performance deficits.

Research using circular arena tasks such as the Morris water maze have been less clear regarding the role of the ATN in spatial behavior. Sutherland and Rodriguez (1989) showed that large lesions of the ATN failed to impair accurate spatial navigation to a hidden platform location. In contrast, Warburton et al. (1999) reported the opposite pattern of results. Further, Stackman et al. (2012) reported that some forms of spatial behavior are retained in the water maze after muscimol inactivation of the ATN. Specifically, impairments were observed in the use of spatial information to guide swim trajectories toward specific directions in the pool, but swim paths toward absolute spatial locations within pool coordinates were seemingly spared. Taken together with the present study, these findings suggest that ATN inactivation is not sufficient to disrupt all aspects of spatial performance in the water maze, but is sufficient to abolish spatial performance in the radial arm maze. An explanation for this pattern of results is the possibility that directional information is required to lesser extent in circular arena tasks, relative to radial maze tasks (Yoder and Kirby, 2014). This might be due to the possibility that “decisions” regarding orientation in the radial maze are executed in the central portion of the maze and paths to goals are confined to maze arms, allowing for no error correction. However, swim trajectories in the Morris water task may be adjusted and re-calibrated along the path to the hidden platform location. Further, evidence suggests that head direction signal disruption has minimal but mixed effects on hippocampal place cell activity in circular environments (Calton et al., 2003), yet has dramatic effects in radial environments such as place field repetition between multiple directionally separated compartments (Wood et al., 2016). This differential involvement of the head direction signal in place cell activity between circular and radial environments suggests that maze geometry can influence the interaction between spatial representations and spatial behavior.

The mechanism by which the ATN may serve a role in spatial memory in the radial arm maze is poorly understood, but one long standing hypothesis has argued that the ATN and head direction cell activity plays a role in the establishment of spatial representations in the hippocampal formation (McNaughton et al., 1991; Sharp et al., 2001; Yoganarasimha et al., 2006; Taube, 2007). Certainly, the fact that lesions or inactivation of the ATN abolish parahippocampal grid cells (Winter et al., 2015) seems confirmatory. In recent work, the ATN head direction cell activity has also been linked to the unique spatial firing patterns of place cells and grid cells in radially oriented environments (Fuhs et al., 2005; Derdikman et al., 2009; Spiers et al., 2015; Grieves et al., 2016). Specifically, the ATN may play a central role in disambiguating spatial locations based on directional orientation (Stackman et al., 2012; Clark et al., 2015; Grieves et al., 2016; Sanchez et al., 2016), which would ultimately influence the accuracy of spatial navigation in the radial maze. Observations of increased activity dependent gene expression in the ATN following training in the radial arm maze supports this hypothesis (Vann et al., 2000), but the effects of direct manipulations of the ATN on hippocampal spatial representation in radial environments is presently unknown.

A final conclusion from the present study relates to the observation that rats with muscimol inactivation of the ATN continued to exhibit exploratory door checking behaviors in the radial maze (Figure 3F). This behavior, which is similar to vicarious trial-and-error (Tolman, 1939; Brown and Cook, 1986; Redish, 2016), has long been argued to serve a role in gathering environmental information, perhaps about the locations of relevant landmarks, and the establishment of spatial representations (O'Keefe and Nadel, 1978). Support for this notion comes from studies demonstrating that these head movements can be altered in rats after hippocampal lesions (Clark et al., 2005; Lehmann et al., 2007; Bett et al., 2015), and that declines in head movements after hippocampal damage can be correlated with spatial learning impairments (Hu and Amsel, 1995). Because the ATN has large reciprocal connections with the hippocampal formation, and contributes to the processing of spatial representations within the hippocampus, a reasonable hypothesis would be that the ATN may also contribute to the guidance of investigatory movements. Nonetheless, the lack of significant changes in door exploration after ATN inactivation in the present study fails to confirm this hypothesis. Further, our findings suggest that deficits in spatial reference memory and spatial working memory after ATN disruption are not explained by alterations in head scanning behaviors and point to a potential functional dissociation between the hippocampal formation and ATN.

To summarize, the results of the present study indicate that the ATN are necessary for spatial discrimination in the radial arm maze as indicated by significant declines in measures of spatial reference memory, spatial working memory, and the use of spatial strategies. Together, the results suggest that the ATN modulates not only the online guidance of accurate spatial behavior, but also appears to be necessary for the expression of previously acquired spatial representations in radial environments. This information adds to a growing body of literature elucidating the role of the head direction signal in navigation.

## Ethics statement

This study was carried out in accordance with the recommendations of the Institutional Animal Care and Use Committee at the University of New Mexico. The protocol was approved by the Institutional Animal Care and Use Committee.

## Author contributions

BC, RY, and RH developed the conceptual framework of the experiment. RH, ST, and LS preformed surgical implantations and collected the data. RH analyzed the data. BC and RH wrote the first several drafts as RY made several edits along the way and BC, RY, and RH wrote the final draft.

### Conflict of interest statement

The authors declare that the research was conducted in the absence of any commercial or financial relationships that could be construed as a potential conflict of interest.
